# Replacement fibrosis detected by CMR is a predictor of outcome in patients with non-ischemic cardiomyopathy

**DOI:** 10.1186/1532-429X-16-S1-P318

**Published:** 2014-01-16

**Authors:** Mohamad G Ghosn, Itamar Birnbaum, Kongkiat Chaikriangkrai, Nadim Nasir, William Zoghbi, Miguel A Quinones, Dipan J Shah

**Affiliations:** 1 Houston Methodist DeBakey Heart & Vascular Center, Houston Methodist Research Institute, Houston, Texas, USA; 2 Cardiology, Houston Methodist Research Institute, Houston, Texas, USA; 3 Medicine, Houston Methodist Hospital, Houston, Texas, USA; 4 Medicine, Baylor College of Medicine, Houston, Texas, USA

## Background

Left ventricular (LV) ejection fraction (EF) is important predictor of clinical outcomes in patients with non-ischemic cardiomyopathy (NICMP). With the advent of gadolinium-enhanced cardiac magnetic resonance imaging (CMR), it has now become possible to discern the presence of myocardial replacement fibrosis (RF) with a high level of accuracy. The aim of our study is to investigate the predictive value of RF in patients with NICMP.

## Methods

A prospective, longitudinal study of the prognostic value of replacement fibrosis in a cohort of 240 consecutive patients with NICMP was performed. The presence and extent of late gadolinium enhancement (LGE) in tissue, which is assumed to represent replacement fibrosis, was determined by visual inspection using the American Heart Association 17-segment model without knowledge of any other clinical factors. Cine-CMR images were also analyzed to calculate functional parameters of the heart (ejection fraction, volumes, etc.). Baseline characteristics were collected and outcome data and clinical events were determined by telephone follow-up, chart review and/or the social security death index.

## Results

The age of our patients was 55.5 ± 15.9 years while males comprised 60% of the population. LVEF was 33.4 ± 10.5%. The median (interquartile range) left ventricular fibrosis extent was 2.0%. There were 42 (17.5%) patients with significant fibrosis (SiF; left ventricular fibrosis > 5%). Patients with SiF were more likely to have history of dyslipidemia (p = 0.005) and smoking (p = 0.004). There were 32 (13%) deaths during follow-up period. Patients with SiF had more death than those without (26%.vs.11%, p = 0.007). Patients who died were more likely to have a history of diabetes (p = 0.007). Both presence of SiF and fibrosis extent were independent predictors for all-cause mortality (p = 0.006 and p < 0.001 respectively). Other independent predictors were history of diabetes (p = 0.006) and BMI (p = 0.006). As secondary clinical outcome, composite of all-cause mortality and heart transplant occurred in 15.4% of the cohort. There were significantly more events in patients with SiF than those without SiF (29%.vs.13%, p = 0.009). Prevalence of history of diabetes, extent of LV fibrosis and LVSV were significantly higher in patients who had reached the secondary outcomes compared to those who did not (p = 0.007, 0.035, 0.042 respectively).

## Conclusions

In patients with NICMP, the presence of scar is an independent risk factor for all-cause mortality and the extent of scar portends a worse prognosis. LGE on CMR provides useful diagnostic information in addition to traditional risk factors of mortality in heart failure and may be relevant to the clinical management of these patients.

## Funding

None.

